# Efficacy and safety of heparin in patients with sepsis: a systematic review and meta-analysis

**DOI:** 10.1186/cc14203

**Published:** 2015-03-16

**Authors:** R Zarychanski, AM Abou-Setta, S Kanji, AF Turgeon, A Kumar, DS Houston, E Rimmer, BL Houston, L McIntyre, AE Fox-Robichaud, PC Hebert, DJ Cook, DA Fergusson

**Affiliations:** 1 University of Manitoba, Winnipeg, MB, Canada; 2 Ottawa Hospital Research Institute, Ottawa, ON, Canada; 3 Université Laval, Québec City, QC, Canada; 4 University of Toronto, ON, Canada; 5 McMaster University, Hamilton, ON, Canada; 6 Centre hospitalier de l'Universite de Montreal (CHUM) - Hopital Notre-Dame, Montreal, QC, Canada

## Introduction

Septic shock is characterized by systemic inflammation coupled with upregulation of coagulation. Heparin is an inexpensive and widely available anticoagulant with anti-inflammatory properties. The objectives our study were to evaluate the efficacy and safety of heparin in patients with sepsis, septic shock or disseminated intravascular coagulation (DIC) associated with infection.

## Methods

We included randomized controlled trials from MEDLINE, EMBASE, CENTRAL, Global Health, Scopus, Web of Science, the International Clinical Trials Registry Platform (inception to April 2014), conference proceedings, and reference lists of relevant articles. Two reviewers independently identified and extracted trial-level data from randomized trials investigating unfractionated or low molecular heparin administered to patients with sepsis, severe sepsis, septic shock or DIC associated with infection. Internal validity was assessed in duplicate using the Risk of Bias tool. Our primary outcome was mortality. Safety outcomes included hemorrhage, transfusion and thrombocytopenia.

## Results

We included nine trials enrolling 2,637 patients. Eight trials were of unclear risk of bias and one was classified as having low risk of bias. In trials comparing heparin with placebo or usual care, the risk ratio for death associated with heparin was 0.88 (95% CI = 0.77 to 1.00, *I*^2 ^= 0%, 2477 patients, six trials). In trials comparing heparin with other anticoagulants, the risk ratio for death was 1.30 (95% CI = 0.78 to 2.18, *I*^2 ^= 0%, 160 patients, three trials). In trials comparing heparin with placebo or usual care, major hemorrhage was not statistically significantly increased (risk ratio 0.79, 95% CI = 0.53 to 1.17, *I*^2 ^= 0%, 2,392 patients, three trials). In one small trial of heparin compared with other anticoagulants, the risk of major hemorrhage was significantly increased (2.14, 95% CI = 1.07 to 4.30, 48 patients). Important secondary and safety outcomes, including minor bleeding, were sparsely reported.

## Conclusion

Heparin in patients with sepsis, septic shock, and DIC associated with infection may be associated with decreased mortality; however, the overall impact remains uncertain. Safety outcomes have been under-reported and require further study. Large randomized trials are needed to evaluate the efficacy and safety of heparin in patients with sepsis, severe sepsis, and septic shock.

